# Has Child Restraint System Use Increased among Parents of Children in Shantou, China?

**DOI:** 10.3390/ijerph13100964

**Published:** 2016-09-28

**Authors:** Huiqian Lei, Jingzhen Yang, Xiangxiang Liu, Xiaojun Chen, Liping Li

**Affiliations:** 1 Injury Prevention Research Center, Medical College of Shantou University, 22 Xin Ling Road, Shantou 515041, China; yufangyixuelhq@sina.com (H.L.); xxliu2014@126.com (X.L.); 2 Center for Injury Research and Policy, The Ohio State University, Columbus, OH 43205, USA; Ginger.Yang@nationwidechildrens.org; 3 The First Affiliated Hospital of Shantou University Medical College, Shantou 515041, China; xjchen@stu.edu.cn

**Keywords:** child restraint system (CRS), attitude, knowledge, parents

## Abstract

Objective: to examine parents’ use of child restraint systems (CRS), and determine if parents’ knowledge of, attitude toward, and use behavior of child restraint systems have improved following enactment of child restraint use laws in other cities. Design: Observations and a cross-sectional survey of drivers transporting children 17 years and under were conducted at the gate of the schools and parking lots of hospitals in Shantou. Observers recorded the seating location of child passengers, the type of restraint, and appropriate use of CRS and safety belts based on the observation. Knowledge of and attitudes towards use of CRS were reported by the driver following observation. Results: Approximately 6.6% of passengers aged 0–12 were in CRS; rate of forward-facing CRS in children aged 3–5 (9.9%) was higher than rear-facing CRS for children aged 0–2 (1.1%) and booster seat use among children aged 6–12 (0.1%). Children younger than four years old (OR = 3.395, 95% CI = 2.125–5.424), drivers having a college or higher lever education (OR = 2.908, 95% CI = 1.878–4.500) and drivers wearing seatbelt (OR = 3.194, 95% CI = 1.605–6.356) had greater odds of CRS use. Over half (56.6%) of parents might or would use CRS if they could rent CRSs with fees. Conclusions: The rate of CRS is still low in Shantou. Comprehensive public education programs supported by legislation might be an effective way to improve child passenger safety. Renting CRSs to parents could be a new approach to encourage use.

## 1. Introduction

Road traffic injuries cause 1.2 million deaths each year, which has serious impact on health and costs governments at approximately 3% of GDP [1]. Road traffic crashes have resulted in almost 50,000 injuries and deaths annually among children aged 0–4 and they are the second leading cause of injuries and deaths for children aged 5 to 14 [2]. The World Health Organization estimates that road crashes result in more than 260,000 deaths in road users every year in China [1]. The number of deaths is expected to increase to half a million per year by 2020 without intervention, and at least 10% of these deaths are children, including child passengers [3].

Traffic-related injuries and deaths are largely preventable. The child restraint system (CRS) is an effective prevention measure in protecting children from injuries and deaths. Existing evidence shows that, when correctly installed and used, CRS reduces the risk of infant fatalities by 71% and toddler fatalities by 54% [4,5].

Despite the strong supporting evidence for CRS use, the rate of CRS use is very low in China. The results from our previous observation study conducted in 2012 among 3333 children in the city of Shantou showed that only 0.4% children aged 0–3 were observed using a rear-facing or front-facing restraint system and 1.1% children aged 4–6 used a CRS [6]. To address the low usage, in 2014, two large cities in China (i.e., Shenzhen and Shanghai) enacted local legislation, which requires child restraint use for children younger than four years old. If children are not restrained, drivers will receive monetary penalties [7,8]. Following enactment of the law, significantly more media reports and local news focused on the importance and benefits of CRS. However, it remains unclear whether the enactment of the child restraint use law in these two cities would help shape the child passenger safety culture in China. Specifically, whether knowledge, attitudes, and behaviors of CRS use among parents living in Shantou (a medium-to-small sized city neighboring Shenzhen) may be influenced by the child restraint use law is unknown. From a Social Ecological Framework perspective, individuals (e.g., parents/caregivers) are embedded within larger social systems (e.g., child passenger restraint law) and the interactive characteristics of individuals (e.g., knowledge of and attitudes towards CRS) and environments (e.g., passenger safety culture in China) will affect health-related behavior—in this case, caregivers’ CRS use behavior [9]. Results from our previous studies, along with those of others, show that parents or caregivers who are more knowledgeable about child passenger safety are more likely to use CRS [8,10,11]. Parents who perceived CRS as necessary to protect child passengers from injury, and who consider legislative requirement for CRS use as needed are associated with a higher rate of CRS use [12,13]. In this study, we replicated the methods used in the observation study conducted in 2012, and aimed to examine parents’ use of CRS, and determine if parents’ knowledge of, attitude toward, and use behavior of child restraint improved following enactment of child restraint use laws in other cities. We hope that the results of this study will provide evidence to support the enactment of local and national laws to protect child passengers.

## 2. Designs

### 2.1. Study Design and Data Collection

An observation study was conducted among drivers who transported children 17 years and younger in Shantou between November and December 2015. Four types of locations were selected representing four age groups of children: immunization clinics of hospitals, including infants and toddlers aged younger than 2 years; kindergartens, comprising children aged 3–5 years; primary schools, containing children aged 6–12 years; and middle schools, including children aged 13–17 years. A total of 4 immunization clinics, 32 kindergartens, 30 primary schools, and 27 middle schools were observed and included in the analysis. We selected the schools and clinics randomly by the locations (north, central, and south) and administrative areas (Longhu region and Jinping region) of Shantou.

A total of 18 observers from the Medical College of Shantou University were recruited to conduct observation and survey data collection. To ensure that the methods of observation and data collection were consistent during the study period, we trained the observers uniformly. The training included showing the observers different types of CRS, providing a script on how to invite potential participants and detailed steps on when, where, and how to conduct observation and/or administer a survey. After training, the observers were given an opportunity to practice these procedures before taking a written test and also a field test. The observers were required to pass both written and field tests before they could conduct data collection for this study. Since all the observation items were based on objective observation, and did not involve subjective rating, we did not assess the inter-rater agreement; rather, we used passing the field test (100% correct) to ensure quality of data collection.

At each observation location, two trained observers conducted each of the observations on child passengers either at the gate of the schools when they were picked up or dropped off or at the hospital parking lot when they visited their doctor to get vaccinations. Information recorded included the driver’s gender, child passenger’s gender, child’s vehicle seating position (e.g., passenger seat, left-back seat, middle-back seat, right-back seat, or adult’s lap) types of restraint (e.g., rear-facing child restraint, forward-facing child restraint, booster, or seat belt), and whether the safety belt was used by the driver appropriately. Additionally, the drivers who transported the child were invited to participate in a brief self-designed in-person survey following the observation regarding their knowledge of and attitude toward CRS. The survey questions included whether it was necessary to use CRS for child passengers, at what ages CRS should be for child passengers, source(s) parents/caregivers learned about CRS, reason(s) why parents were not using CRS, whether parents would use CRS if provided at a charge, and whether parents would use CRS if provided for free. The study procedure was approved by the Ethics Committee of the Medical College, Shantou University (Code: SUMC-2015-38).

### 2.2. Statistical Analysis

Data collected were analyzed using SPSS version 21.0 (SPSS Inc., Chicago, IL, USA). Descriptive analysis was used to describe child restraint use and parents’ knowledge and attitude towards CRS. Differences in children’s riding position, restraint use, as well as type of CRS use were compared by different age groups using Chi-square tests. The parents’ knowledge and attitude towards CRS were compared between CRS use and CRS non-use groups using Chi-square tests. The demographic factors and parents’ knowledge and attitudes associated with CRS use were assessed using unadjusted and adjusted logistic regressions. The factors associated with CRS use were reported as OR and 95% CI. Since 18 chi-squared tests were conducted in this study, Bonferroni correction method was used to calculate the adjust *p*-value for significant tests to control family-wise error. Thus, *p*-value less than 0.0028 (0.05/18) was considered as statistically significant.

## 3. Results

### 3.1. Observations

A total of 3464 child passengers (including 586 infants and toddlers, 1221 kindergarteners, 1043 pupils, and 614 teenagers) were observed. As seen in Table 1, more than 85% of passengers observed in the study did not use any restraints when they were riding in the cars. There were significant differences in restraint used in different ages (*p* = 0.002), with a higher proportion of restraint use among children 13–17 years old (18.7%), followed by those 3–5 years old (15.3%). Approximately 6.6% (188/2850) of passengers aged 0–12 were in CRS, while only 1.1% (*n* = 6) of infants and toddlers were using age-appropriate rear-facing CRS (younger than age 24 months or not exceeding 30 pounds for forward-facing child seats) and about 10% (121/1043) of kindergarteners were using forward-facing CRS. Only 0.1% (*n* = 1) of children aged 6–12 were using boosters as recommended, and 10.9% of them were using seat belts, which is not appropriate. There were significant differences in types of restraints used among children aged 0–12 years old (*p* < 0.001). About 18% of children were in the front seat and 10% were sitting in an adult’s lap being held by an adult. The proportion of children sitting in an adult’s lap was particularly high (37.4%) for passengers ages two or younger.

### 3.2. Surveys

As seen in Table 2, nearly one-third (*n* = 1112, 32.1%) of drivers participated in the brief survey, with 1003 drivers having children under age 12 completing this survey. When asked “Is it necessary to use CRS for child passengers?” among all respondents, 94.5% said “Yes”, but the rate of actual use was less than 7.0%. Most of the respondents (85.9%) indicated that child passengers ages 2 to 5 should use CRS, while 62.7% and 30.6% of respondents indicated that child passengers ages 0 to 1 or 6 to 12 should use CRS, which was inconsistent with observed actual use of CRS. Nearly 80% (79.1%) of respondents learned about CRS from media (television, internet, newspaper, magazine). About 30% (30.9%) of respondents learned about CRS because their relatives and friends’ recommendations, and 15.0% learned about CRS because of enactment of child restraint use legislation in other cities. When asking respondents about the reasons for not using CRS, 44.0% reported no law requirement, 29.3% said inconvenience, and 19.9% said because of the high price. Over 20% (22.4%) of parents considered it safer to hold child passengers by themselves, especially for younger children. There were significant associations between CRS use and parents’ knowledge of and attitudes towards use of CRS. Specifically, parents who reported CRS use as necessary had increased CRS use (*p* < 0.01). Over 70% (70.1%) of respondents said they might or would use CRS if free of charge, but only 55.4% said they might or would use it if they needed to pay a rental charge.

Table 3 presented the unadjusted and adjusted Odds Ratios (ORs) of CRS use based on logistic regression analyses. Child’s age, driver’s age, driver’s seatbelt use, and driver’s education were significantly associated with CRS use. Results from adjusted logistic regression showed that children younger than four years old (OR = 3.395, 95% CI = 2.125–5.424), drivers having a college or higher lever education (OR = 2.908, 95% CI = 1.878–4.500), and drivers wearing seatbelt (OR = 3.194, 95% CI = 1.605–6.356) had greater odds of use CRS.

## 4. Discussion

Although the rate of CRS use of those younger than 12 was substantially increased compared to 2012 [13], this study showed CRS use in Shantou to still be very low. Compared with rates of CRS use among children ages 0–5, the usage among children 6–12 years old was unacceptably low, with a rate of 0.1%. On the one hand, many parents of children ages 6 to 12 consider their child to be old enough to wear seat belts, thus, they choose not to install boosters. It is possible these parents are unaware that children who transition to seat belts too early could be at increased risk of crash-related injuries including spinal injury [14]. On the other hand, other cities require child restraint use for children younger than four years old may have helped increase CRS use behaviors among parents of this age group. It should be noted that, although the rate of CRS use among children aged 0–5 was increasing, the rate is still low; it remains especially low among infants and toddlers. It is lower than the United States [15], Australia [16], and Japan [17], and even lower than Pakistan [12] compared with other countries, and lower than Shenzhen, Beijing, and Shanghai compared with other cities in China [7,8,18]. There are likely several reasons for low rear facing CRS use. First, some parents might think their babies are too young to be in rear facing restraint system or they believe holding the children by themselves are safer than in CRS [10]. Second, overly emphasizing the forward facing restraint system in Chinese media may result in parents’ misconceptions regarding the importance of the rear facing and booster seats [13]; Lastly, some parents reported they knew rear facing was safer for children 0–2 years old, but still placed the child forward facing because they mistakenly believed the forward facing CRS would last longer and be more cost-effective [19,20].

Our results showed that nearly half of respondents reported they did not use CRS for their child passengers because of no law requirement. Our results were supported by previous findings, which showed that legislation can encourage parents’ use CRS in China [7,8], suggesting the legislation is likely an effective intervention to improve child passenger safety. Previous studies show that awareness of child passenger safety is closely related to the education provided by the government [16,17,21,22,23]. Mass media has become a major channel to gain knowledge of child passenger safety for most parents, especially new and popular media channels such as We-chat and micro-blogs. To improve parents’ CRS behaviors, public education programs may consider incorporating these new media channels to help parents understand the importance of CRS and how to properly use different types of based on the age and characteristics of the child.

Parents, for the most part, agree it is necessary to install a restraint system for their child passengers, but the majority of them failed to take further actions to use CRS. It is evident from these findings that awareness and intention do not equate to action. This discrepancy was also reported in study in Portugal which 97.3% intended to use the CRS, however only 47.3% would do it adequately with parents lacking knowledge in the use of CRS [24]. In Saudi Arabia, a separate behavioral observation showed that intentions might not lead to the actual usage of CRS in pregnant women [25]. It indicated that, without fully understanding the critical importance and mastering the knowledge of CRS, the care-givers may fail to use CRS and protect the child passenger safety. CRS use was significantly associated with wearing the seat belt by the driver. Our finding was supported by the study conducted on 594 Northwest American India drivers which found drivers’ wearing a seatbelt were more likely to use CRS (OR = 2.39; 95% CI = 1.51, 3.80) [26]. Our finding was also supported by the studies conducted in Brazil [27] and Nigeria [28]. It is not surprising, as drivers’ use of seatbelts implies that they have higher safety conscious behavior and are more likely to ensure the safety of their passengers. According to the Global Statue Report on Road Safety [1] in China, the enforcement level of national seat-belt law is 8 (total 0–10 levels; the higher the level, the stronger the enforcement). Effective prompt awareness of child passenger safety could shield light on how seat-belt laws can be enhanced in China.

A high proportion of parents did not use CRS due to the high price and short-period of use. Over half of parents reported they might or would accept CRS by paying a charge. This revealed a possibility of a new rental market which provides the parents with applicable CRS to encourage and increase the use and at the same time reduce the financial burden if the law requires CRS. In Greece, one hospital attempted to rent CRSes to parents of newborns for six months. It showed that 92% of parents installed and used CRS correctly during the lease term and 82% of them bought the next stage of CRS. Further research is needed to evaluate the potential of CRS rental strategy if CRS use is required by law.

## 5. Limitations

This study has several limitations. First, the results of the present research may not be generalized to represent the overall population of China. Shantou, while in medium-to-small-sized, is located in one of the most economically developed regions of China, which is significantly different from cities in the more inland regions of China, especially regarding population income level and ownership of passenger cars. Second, the findings of this study may be affected by reporting bias. Due to social desirability, parents may have been more likely to report that CRS installation was necessary. Last, researchers were at the gates of schools for observation. A few respondents reported that they only use the CRS while driving on the freeway with a child, but not when they pick-up or drop-off the child. In this case, our results of observation in the gates of schools may underestimate CRS use. Observation conducted at highway stations may have interesting findings in a further study.

## 6. Conclusions

The rate of CRS use is still low in Shantou. Comprehensive public education programs supported by legislation might be an effective way to improve child passenger safety. Renting CRSes to parents could be a new approach to encourage use.

## Figures and Tables

**Table 1:** Observed child riding positions and restraint use of child passengers by age group (*n*/%).

		Age Group				
	Total	Immunization Clinics Children Aged 0–2	Kindergarten Children Aged 3–5	Primary School Children Aged 6–12	Middle Schools Children Aged 13–17	*p*-Value
Total	3464	586	1221	1043	614	
Riding position						<0.0001
Front seat	630	24	203	199	204	
	18.2%	4.1%	16.6%	19.1%	33.2%	
Rear seat	2515	343	948	817	407	
	72.6%	58.5%	77.7%	78.3%	66.3%	
Adult’s lap	319	219	70	27	3	
	9.2%	37.4%	5.7%	2.6%	0.5%	
Restraint used ^a^						0.0018
Yes	505	76	187	127	115	
	14.6%	13.0%	15.3%	12.2%	18.7%	
No	2959	510	1034	916	499	
	85.4%	87.0%	84.7%	87.8%	81.3%	
Type of restraint used						<0.0001 ^b^
Rear-facing child restraint	10	6	3	1	0	
	0.3%	1.1%	0.2%	0.1%	0	
Forward-facing child restraint	175	43	121	11	0	
	5.1%	7.3%	9.9%	1.1%	0	
Booster	4	1	1	1	1	
	0.1%	0.2%	0.1%	0.1%	0.1%	
Seat belt	316	26	62	114	114	
	9.1%	4.4%	5.1%	10.9%	18.6%	

^a^ Including CRS (rear-facing child restraint, forward-facing child restraint and booster) and seat belt; ^b^ Excluding the age group of 13–17, use the Fisher’s exact probability.

**Table 2:** Attitudes and knowledge on CRS of surveyed drivers ^a^.

	CRS Used			
Items	N (1003)	Yes (141)	No (862)	*p-*Value
Use CRS				0.0045 ^b^
Necessary	956 (95.3%)	141 (100.0%) ^c^	815 (94.5%)	
Unnecessary	47 (4.7%)	0	47 (5.5%)	
Ages of use of CRS ^c^				0.0270
0–1	629 (62.7%)	110 (78.6%)	519 (60.2%)	
2–5	862 (85.9%)	133 (95.0%)	729 (84.6%)	
6–12	307 (30.6%)	63 (45.0%)	244 (28.3%)	
13–17	17 (1.7%)	7 (5.0%)	10 (1.2%)	
Sources of learning about CRS ^c^				<0.0001
Media	793 (79.1%)	117 (83.0%)	676 (78.4%)	
Family or friend recommendation	310 (30.9%)	53 (37.6%)	256 (29.7%)	
Impact of legislation	150 (15.0%)	39 (27.7%)	111 (12.9%)	
Have not learned	69 (6.9%)	1 (0.7%)	69 (8.0%)	
Other	56 (5.1%)	7 (5.0%)	49 (5.1%)	
Reasons for not using CRS ^c^				0.5950
No law enforcement	441 (44.0%)	79 (56.0%)	362 (42.0%)	
Inconvenient to use	294 (29.3%)	43 (30.5%)	251 (29.1%)	
Lack adequate awareness	244 (24.3%)	41 (29.1%)	203 (23.5%)	
Safer to embrace passengers	225 (22.4%)	30 (21.3%)	195 (22.6%)	
High charge	200 (19.9%)	36 (25.5%)	164 (19.0%)	
Other	184 (18.3%)	27 (19.1%)	157 (18.2%)	
Provide CRS with charge				<0.0001
Refuse	447 (44.6%)	57 (40.4%)	390 (45.2%)	
Might accept	364 (36.3%)	38 (25.5%)	328 (38.1%)	
Accept	192 (19.1%)	48 (34.1%)	144 (16.7%)	
Provide CRS for free				<0.0001
Refuse	300 (29.9%)	35 (24.8%)	265 (30.7%)	
Might accept	340 (33.9%)	34 (24.1%)	306 (35.5%)	
Accept	363 (36.2%)	72 (51.1%)	291 (33.8%)	

^a^ Including the drivers whose children are under the age of 12; ^b^ Use the Fisher’s exact probability; ^c^ Multiple choices, total percentage is more than 100%.

**Table 3:** Unadjusted and adjusted odds of CRS use ^a^.

Variable	CRS Use						
	Yes	No	*p*-Value	Unadjusted OR	95% CI	Adjusted OR	95% CI
Child’s age							
≥5	30	399	<0.0001	1			
≤4	111	462		3.195	2.089–4.888	3.395	2.125–5.424
Child’s gender							
Boy	84	437		1			
Girl	56	417	0.1473	0.707	0.497–1.006	0.757	0.522–1.098
Driver’s age							
≤35	114	613	0.01639	1			
≥36	27	249		0.583	0.374–0.910	0.974	0.573–1.654
Driver’s gender							
Male	67	392	0.6518	1			
Female	74	470		0.921	0.645–1.316	1.009	0.690–1,478
Relationship with child							
Not parent	9	48	0.6990	1			
Parent	132	814		0.865	0.415–1.804	1.105	0.759–1.609
Education							
<college	31	371	<0.0001	1			
≥college	110	491		2.681	1.760–4.084	2.908	1.878–4.500
Driver’s seatbelt use							
No	10	212	<0.0001	1			
Yes	131	650		4.273	2.205–8.249	3.194	1.605–6.356
Provide CRS with charge							
Refuse	57	390	0.2859	1			
Might/would accept	84	472		1.218	0.848–1.749	1.078	0.645–1.799
Provide CRS for free							
Refuse	35	265	0.1547	1			
Might/would accept	106	597		1.344	0.893–2.023	1.040	0.585–1.850

^a^ Including the drivers whose children were under the age of 12.
